# Supplementary material for: Ultrasound-Mediated Lysozyme Microbubbles Targeting NOX4 Knockdown Alleviate Cisplatin-Exposed Cochlear Hair Cell Ototoxicity
Source: Int J Mol Sci. 2024 Jun 28;25(13):7096. doi: 10.3390/ijms25137096 (PMC11241201; doi:10.3390/ijms25137096)
Supplement: Supplementary file 1 [file ijms-25-07096-s001.zip › ijms-3041097-supplementary.pdf]

Supplementary Figure S1:

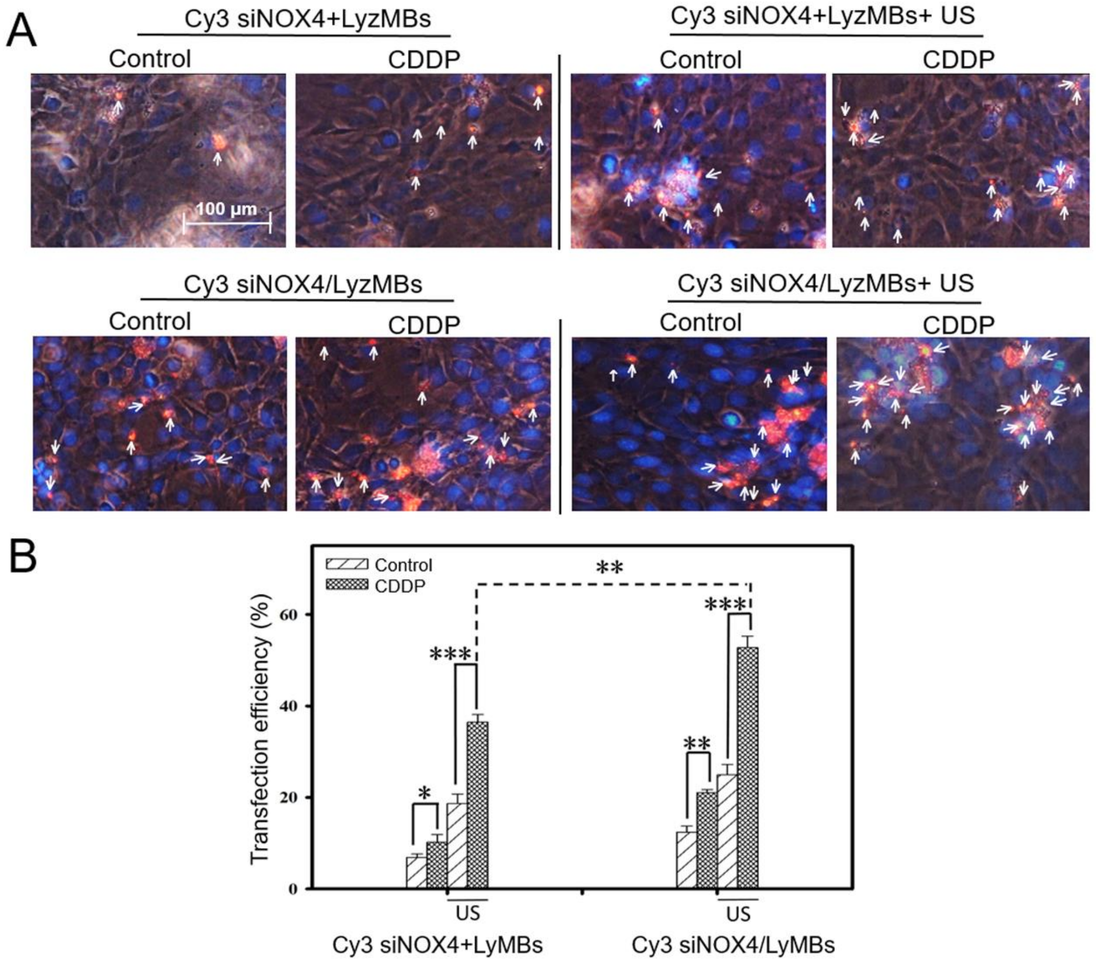

Figure S1: Transfection with siNOX4/LyzMBs before CDDP treatment. Fluorescence microscopy images of Cy3 siNOX4 transfection using it mixed with LyzMBs (Cy3 siNOX4+LyzMBs group) or loaded on LyzMBs (Cy3 siNOX4/LyzMBs group) in HEI-OC1 cells before CDDP or saline (control) treatments, with or without ultrasound (US) sonication (**A**). Quantification of the siNOX4 transfection efficiencies for HEI-OC1 cells (**B**). Scale bar = 100 µm. *$p < 0.05$, **$p < 0.01$, ***$p < 0.001$. Data are expressed as mean ± SEM, with $n = 5$ for each bar.
